# Modified Subclavian Artery-to-Right Atrium Bypass Technique in a Patient on Chronic Hemodialysis With Central Venous Occlusion

**DOI:** 10.7759/cureus.104480

**Published:** 2026-03-01

**Authors:** Yassine Morjane, Adnane Benzirar, Omar El Mahi, Hicham El Malki, El Mehdi Moutaouekkil

**Affiliations:** 1 Department of Cardiovascular Surgery, Mohammed VI University Hospital Center, Oujda, MAR; 2 Department of Vascular Surgery, Mohammed VI University Hospital Center, Oujda, MAR; 3 Faculty of Medicine and Pharmacy, Mohammed First University, Oujda, MAR

**Keywords:** central venous occlusion, chronic hemodialysis, end-stage kidney disease, subclavian artery to right atrium bypass, vascular access

## Abstract

It is not uncommon for all of the usual upper and lower extremity autogenous access sites to fail, often in patients for whom neither peritoneal dialysis nor transplantation is an appropriate option or is difficult to access, and who have chronic central venous occlusion. Right atrium bypass may be used as a last resort. We describe our technique through this clinical report.

The patient is 40 years of age and has a history of type 1 diabetes. The bypass was performed via a minimally invasive approach (right anterior mini-thoracotomy), using a polytetrafluoroethylene (PTFE) prosthesis as a conduit; all procedures were technically successful and resulted in the creation of a new dialysis graft in the thorax.

## Introduction

The permanent loss of vascular access in some patients and the impossibility of carrying out a "conventional" hemodialysis vascular access by native arteriovenous fistula (AVF) or arteriovenous bypass require the implementation of new forms of vascular access, called "exotic," respecting the principle of the AVF [1].

The article reports the technical note of a patient with chronic hemodialysis, who lost all possibility of carrying out a "conventional" hemodialysis vascular access, and in whom an exotic vascular access by subclavian artery to right atrium bypass was performed by the cardiovascular surgery and peripheral vascular surgery teams of the Mohammed VI University Hospital in Oujda [2-4]. To our knowledge, this experience is the first of its kind nationally.

Through this technical report, and in the light of data from the medical literature, the aim of this article is to demonstrate the technical feasibility of this new type of permanent vascular access, its advantages, and its uses.

## Technical report

The patient was 40 years of age and had a history of type 1 diabetes (as early as the age of 16), complicated by bilateral proliferative diabetic retinopathy and diabetic nephropathy since the age of 31, with end-stage kidney disease (ESKD) at the hemodialysis stage after two years (at the rate of three sessions per week).

The onset of the current condition dates back seven years prior to admission, when he was initially dialyzed by a right jugular catheter for a few months, having subsequently benefited from several types of AVF (radiocephalic, humerobasilic, humerocephalic, and femorofemoral), which always end in non-recoverable acute thromboses and become non-functional. Thereafter, hemodialysis was provided by a right tunneled subclavian catheter.

Faced with this therapeutic impasse, following the exhaustion of the vascular capital with no possibility of carrying out a conventional primary or secondary vascular approach, it was decided to perform a central vascular approach by interposing a polytetrafluoroethylene (PTFE) prosthetic tube between the right subclavian artery and the right atrium by a minimally invasive approach.

**Figure 1:**
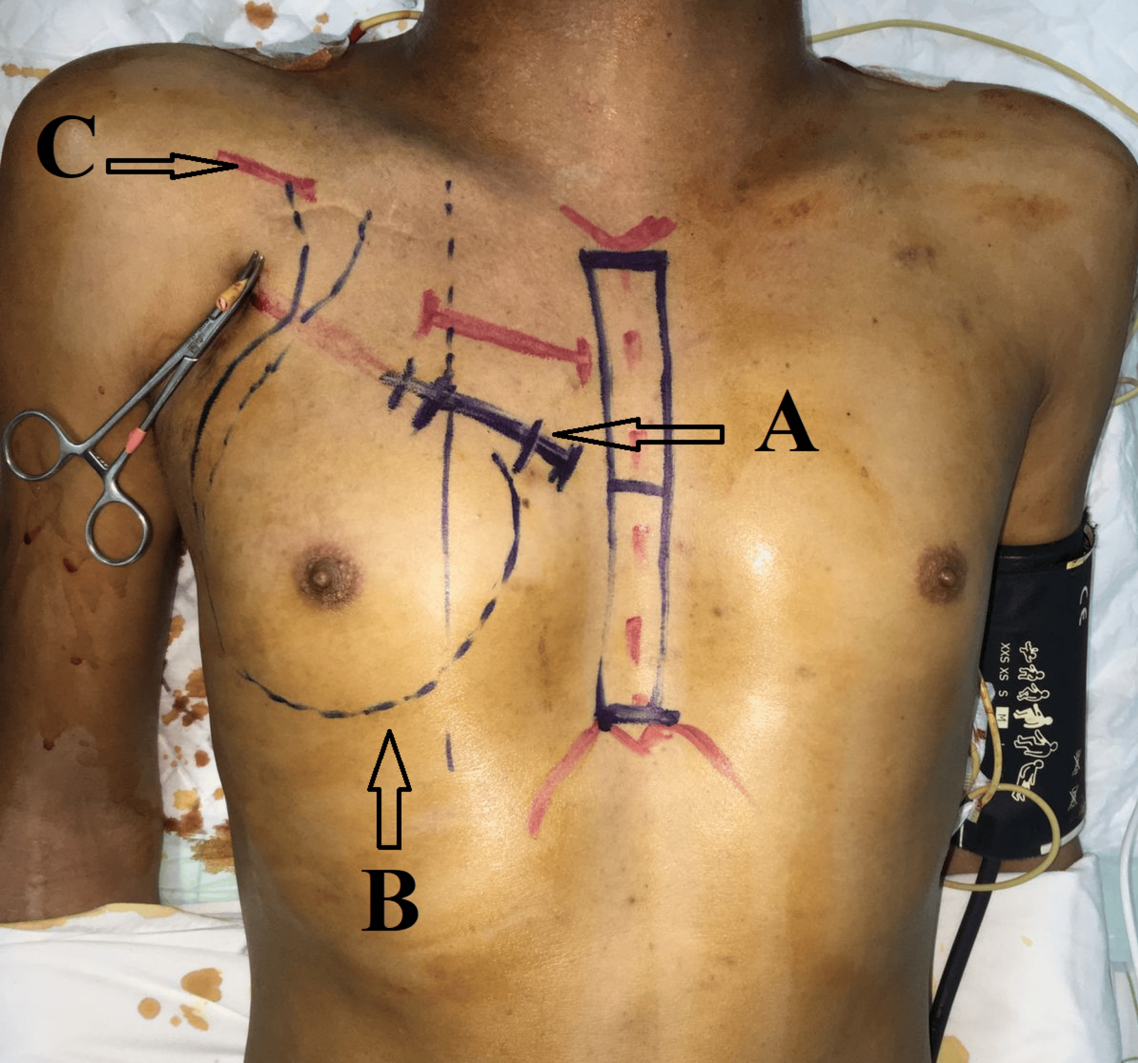
Intraoperative view showing the main anatomical landmarks of a right atrio-subclavian approach. A: right anterior mini-thoracotomy approach; B: the tunneling path of the PTFE vascular prosthesis; C: subclavian approach. PTFE: polytetrafluoroethylene

In the operating room, surgical identification had been carried out after patient positioning; in particular, the surgical approaches to the subclavian artery and the right atrium, as well as the tunneling path of the PTFE vascular prosthesis (Figure 1).

A first approach to the right subclavian artery at the level of the external 2/3 of the right supraclavicular furrow was carried out; this appeared to be of satisfactory quality.

**Figure 2:**
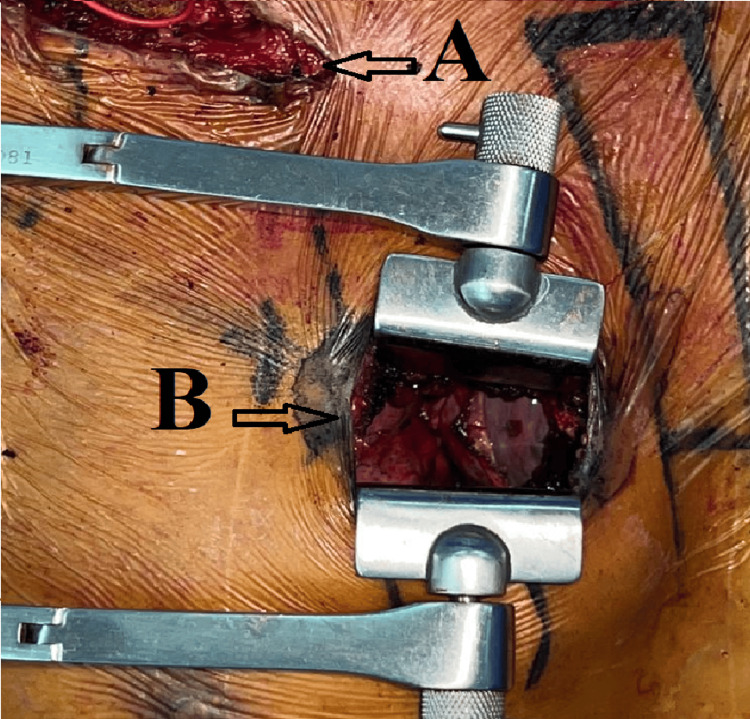
Intraoperative view showing the main surgical approaches. A: the surgical approach to the subclavian artery; B: the surgical approach to the right atrium.

A second approach by an anterior mini-thoracotomy at the level of the second right intercostal space was performed; a section of the internal mammary pedicle between two hemoclips, a disinsertion of the chondrocostal joint of the third rib, and a pericardiotomy in front of the right phrenic nerve with the installation of several wires of suspension made it possible to exert a traction on the pericardium and to better expose the right atrium (Figure 2).

After systemic heparinization (50 IU/kg) and lateral clamping of the right atrium with a Satinsky clamp, an atriotomy of about 1 cm was performed on the lateral face of the right atrium; the pectineus muscle strips were then cut to release the connection of the vascular prosthesis.

**Figure 3:**
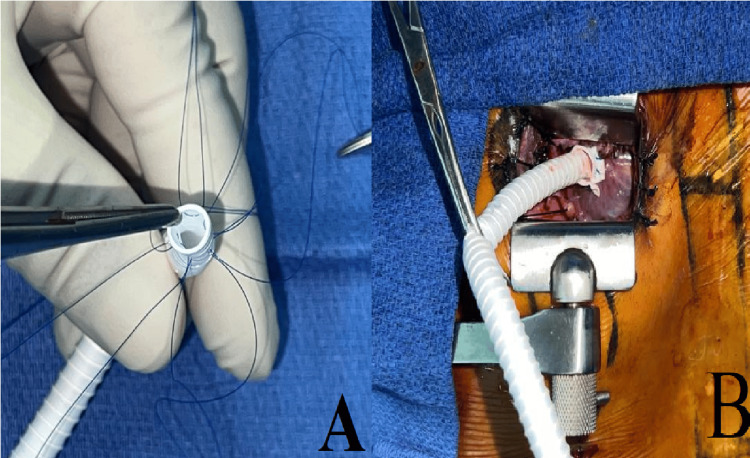
Reinforced tube with inclusion technique using several 5-0 polypropylene sutures. A: assembly of several 5-0 polypropylene sutures on the prosthesis 2 cm from the distal end; B: final aspect of the distal anastomosis with inclusion technique.

Initially, the distal anastomosis was performed using a No. 6 mm PTFE reinforced tube on the right atrium with inclusion of the first two centimeters of the prosthesis inside the right atrium, so that the distal anastomosis would be made 2 cm away from the junction of the prosthesis with several 5-0 polypropylene sutures, then reinforced with a strip of felt (Figure 3A, 3B).

After tunneling the tube through the second intercostal space, then in a subcutaneous loop at the level of the pectoral region to the level of the subclavian groove, an arteriotomy of the subclavian artery was performed, and the proximal anastomosis of the tube was made by a continuous overlock of 5-0 polypropylene suture.

**Figure 4:**
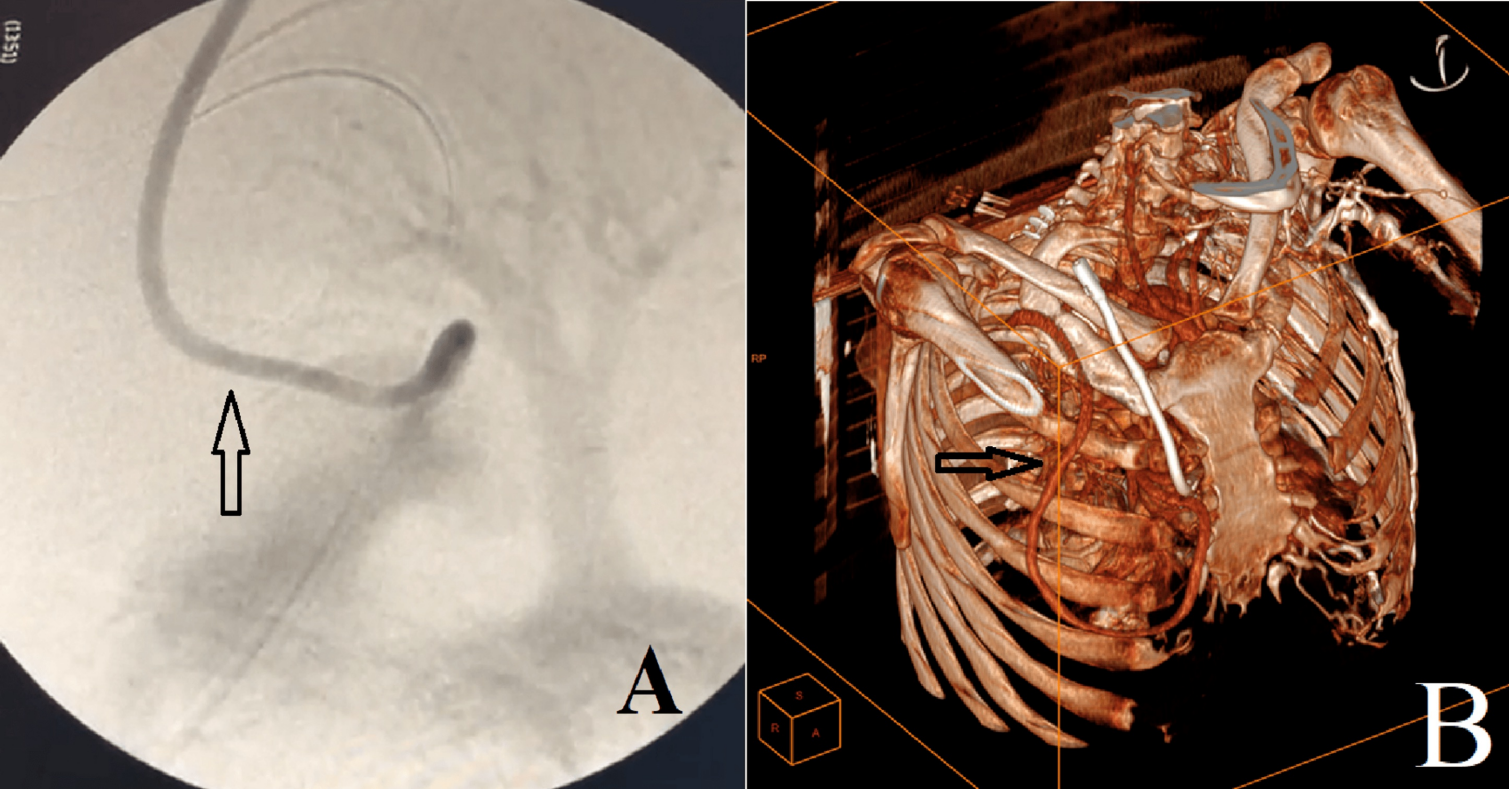
Radiological images showing good flow at the level of the PTFE prosthesis. A: a scopic control at the end of the intervention; B: a reconstruction of a chest angiography one week after the operation. PTFE: polytetrafluoroethylene

Immediately after surgery, a control imaging showed good functioning of the prosthesis (Figure 4A, 4B).

The patient was transferred to intensive care, with curative anticoagulation and antiplatelet therapy. The post-operative follow-ups were simple. An antiplatelet therapy-based treatment was prescribed upon discharge, with regular follow-up at the vascular surgery consultation.

## Discussion

Patients with ESKD at the hemodialysis stage, who have exhausted their peripheral access site, and who also have central venous occlusion, represent a unique therapeutic challenge for nephrologists and vascular surgeons because these are real situations of therapeutic impasses.

Endovascular therapy is the first-line approach for the treatment of central venous occlusion and superior vena cava syndrome (SVCS), but its failure leads to the search for a surgical alternative for the creation of a new vascular approach [1].

In the cases of patients with exhausted upper and lower extremity peripheral veins, new configurations have been reported, using the renal vein [2], inferior vena cava [3], or right atrium as the outlet, although no long-term studies have been reported. Peritoneal dialysis and support for kidney transplantation are encouraged whenever possible to overcome this therapeutic impasse.

In this technical report, we are interested in an approach using the heart as a direct outlet with the creation of a hemodialysis graft from an artery (subclavian [4], axillary [5], brachial [6], or femoral [7]). This graft is tunneled under the skin, which will allow its direct puncture during hemodialysis sessions. As with arteriovenous grafts placed in the peripheral vasculature, this right atrial arterial graft can suffer from thrombotic and stenotic complications.

Concerning the present report, the patient refused peritoneal dialysis, and his anatomical conditions were not favorable to carrying out a vascular approach in the lower limbs.

The preoperative evaluation of the patient was satisfactory for carrying out a central vascular approach. The central arteriovenous bypass was performed using a mini-thoracotomy, which represents an alternative to direct reconstruction through a sternotomy and which offers a satisfactory approach for performing the distal anastomosis on the right atrium, compared to the vertical median sternotomy.

The thoracotomy procedure remains an excellent substitute: it allows the distal anastomosis to be performed in complete safety while reducing the risk of postoperative complications in the population of chronically fragile hemodialysis patients, with, in particular, a reduction in postoperative pain, infections of the incision route, bleeding, transfusions, supraventricular arrhythmias, and consequently, a reduction in the length of the hospital stay [8].

Regarding the technique of distal anastomosis of the prosthesis on the right atrium to reduce the incidence of thrombosis, due to intimal hyperplasia around this anastomosis, we carried out a technique of inclusion of the first two centimeters of the PTFE prosthesis inside the right atrium so that the distal anastomosis will be made 2 cm away from the prosthesis insertion, which would make it possible to eliminate the hemodynamic stress induced by the flow of arterial blood in an intima accustomed to a blood flow at low pressure of the venous type.

The reinforced PTFE prosthesis was chosen because of its availability on the Moroccan market compared to biological grafts (human saphenous vein type or bovine carotid artery), whose long-term permeability seems better [9]. Moreover, the patient does not have autologous veins that can be used for bypass surgery, particularly in the lower limbs.

Reinforced PTFE prostheses offer better anti-thrombogenicity, resistance to extrinsic compression, and relative ease of puncture. They have patency rates comparable to vein grafts when used in conjunction with a distal AVF and perform well in the long term for short reconstructions in the mediastinum [10]. Several studies have reported secondary patency rates greater than 80% at long-term follow-up [11-13].

These exotic bypasses have the added benefit of easy puncture [14]. The other advantage is in cases where the vessels of the arm and forearm are atheromatous, the axillary artery provides better blood flow and generally does not cause vascular theft and therefore ischemia threatening the homolateral upper limb [14].

Regarding the use of anticoagulant and antiplatelet therapy, anticoagulation for three to six months has been recommended, followed by antiplatelet therapy exceptionally indicated in cases of hypercoagulability or in the event of early thrombosis of the bypass (before six months) [13]. Other authors do not recommend any anticoagulation because of the existence of platelet dysfunction in chronic hemodialysis patients [15].

For this patient, we instituted anticoagulation with unfractionated heparin for 24 hours and then antiplatelet aggregation with aspirin at a dose of 75 mg per day.

The monitoring of these vascular approaches was initially done clinically by auscultation through the perception of the thrill, radiologically by arterial echo-Doppler for a week, then reviewed in vascular consultation regularly for three weeks to watch for any dysfunction of the vascular access. The continuity of this monitoring will be ensured by the hemodialysis team, monitoring of the thrill, and monitoring of the reperfusion pressures.

## Conclusions

Maintaining vascular access in patients with ESKD remains a persistent challenge. In particular, central venous occlusion of the superior and inferior vena cava often presents a major complication in these patients. Endovascular treatment, including angioplasty and stenting, is widely regarded as the gold standard for preserving vascular access. However, its long-term patency remains limited, prompting the search for alternative solutions, including more invasive surgical procedures. Subclavian artery to right atrium bypass is one such alternative. Its feasibility can be enhanced within a multidisciplinary framework through collaboration among nephrology, radiology, vascular surgery, and cardiac surgery teams.
